# Supplementary material for: Transfusion Transmissible Infections in Blood Donors in the Province of Bié, Angola, during a 15-Year Follow-Up, Imply the Need for Pathogen Reduction Technologies
Source: Pathogens. 2021 Dec 17;10(12):1633. doi: 10.3390/pathogens10121633 (PMC8705259; doi:10.3390/pathogens10121633)
Supplement: Supplementary file 1 [file pathogens-10-01633-s001.zip › pathogens-1467103-supplementary.pdf]

Table S1. Characteristics of the 500 blood donor samples collected in 2007, and those positive for anti-HCV.

**Total Blood Donors, n**	**500**
Gender	
Male—*n* (%)	412 (82.4)
Female—*n* (%)	88 (17.6)
Age, mean ± SD (range), years	429.3 ±9.2 (18–64)
Age category	
18–24	251 (50.2)
25–49	225(45.0)
>50	13(2.6)
unknown	11(2.2)
Anti-HCV positive *n* (%)	20 (4)
Male—*n* (%)	18 (4.4)
Female—*n* %	2 (2.2)
Age 18–24—*n* (%)	8 (3.1)
25–49—*n* (%)	12 (5.3)
> 50—*n* (%)	0 (0)
HCV RNA testing *n*	20
Detected	0
Not detected	20
Malaria testing, *n* (%)	20
Positive	14 (70)
Negative	6 (30)

The results presented in this table, Supplement 1, are from 500 blood donor in 2007 that consented in providing additional samples that were sent to Brazil for testing. NOTE: the number of blood donors tested for anti-HCV in 2007 displayed in Table 1 and Table 2 (n=848) in the manuscript differ from this table as they refer to data obtained from the *Hospital Geral do Bié´s* blood bank records for year 2007. *n*, total number; SD, standard deviation.

Table S2. List of rapid tests used during the study period.

Commercial Rapid Test	Manufacturer	Sensitivity (%)	Specificity (%)
**HBV**			
Determine™HBsAg[1]	Abbott Laboratories, Tokyo, Japan	98.36	100.00
Vikia®HBsAg[1]	bioMerieux®Brasil SA, Rio de Janeiro, Brazil	98.92	99.79
Advanced Quality™ One Step HBsAg test[1]	InTec Products, Inc., Fujian, China	98.87	98.89
**HCV**			
SD Bioline™HCV[2]	Standard Diagnostics, Kyounggi-do, Korea	99.30	100.00
Vikia® ANTI HCV[3]	bioMerieux®SA, Marcy l'Étoile, France	99.62	99.90
Anti-HCV Spot[4]	Genelabs Diagnostics Pty Ltd, Singapore	97.3	100.00
HCV rapid test Bioeasy™ [1]	Bioeasy Diagnostica Ltda.	100.00	99.40
Rapid Signal™ HCV[1]	Orgenics Ltda., Yavne, Israel	99.00	98.60
**HIV**			
Determine™ HIV 1/2[1]	Abbott Laboratories, Tokyo, Japan	99.00	100.00
Unigold™ HIV[1]	Trinity Biotech, Dublin, Ireland	100.00	100.00
Vikia® HIV1/2[1]	bioMerieux®Brasil SA, Rio de Janeiro, Brazil	99.86	99.95
Teste rápido HIV 1/2 Cypress™ [1]	Cypress Diagnostics, Langdorp, Belgium	100.00	99.50
**Syphilis**			
RPR-Carbon[5]	Biotech	100.00	98.80
VDRL[6]	Atlas Medical	78.00	98.00
Alere Determine™ Syphilis[7]	Abbott Laboratories, Norwich Connecticut, USA	97.20	94.10
Teste rapide de la Syphilis Cypress™[1]	Cypress Diagnostics, Langdorp, Belgium	99.30	99.50
Syphilis Strip Test Bioeasy™ [1]	Bioeasy Diagnostica Ltda., Minas Gerias, Brazil	99.00	99.50

Sensitivity and specificity obtained through rapid test inserts. [1] manufacturers package insert;

[2] manufacturers website https://maxanim.com/content/abbott/sd-bioline-hcv.pdf;

[3] https://www.biomerieux.co.uk/sites/subsidiary_uk/files/vikia_anti-hcv_brochure.pdf;

[4] World Health Organization Report http://apps.who.int/iris/bitstream/handle/10665/66829/WHO_BCT_BTS_01.2.pdf?sequence=1;

[5] https://www.info.com/serp?q=sifilis+RPR+Carbon&sc=CpYU4oQWsco410#:~:text=https%3A//pt.scribd.com/document/495792849/RPR-carbon;

[6] https://atlas-medical.com/upload/productFiles/202002/PPI1535A01%20VDRL%20Ag%20kit%20with%20(+ve,ve)%20Rev%20A.pdf; [7] https://www.info.com/serp?q=TESTE+RAPIDO+DETERMINE+PARA+SIFILIS&sc=T0RKVn9QdY4D10#:~:text=https%3A//doctorlab.com.br/produtos/alere-determine-syphilis-100t-teste-de-sifilis.
